# All for One: The Role of Colony Morphology in Bryophyte Desiccation Tolerance

**DOI:** 10.3389/fpls.2019.01360

**Published:** 2019-11-12

**Authors:** Ricardo Cruz de Carvalho, António Maurício, Manuel Franscisco Pereira, Jorge Marques da Silva, Cristina Branquinho

**Affiliations:** ^1^cE3c, Centre for Ecology, Evolution and Environmental Changes, Faculdade de Ciências, Universidade de Lisboa, Lisbon, Portugal; ^2^MARE—Marine and Environmental Sciences Centre, Faculdade de Ciências, Universidade de Lisboa, Lisbon, Portugal; ^3^CERENA, Instituto Superior Técnico (IST), Universidade de Lisboa, Lisbon, Portugal; ^4^BioISI, Biosystems and Integrative Sciences Institute and Departamento de Biologia Vegetal, Faculdade de Ciências, Universidade de Lisboa, Lisbon, Portugal

**Keywords:** bryophyte, morphology, x-ray computed microtomography, desiccation, water retention

## Abstract

In the last decade, several works showed that even bryophytes from aquatic environments, if slowly dehydrated, can cope with desiccation in a response like the one from desert bryophytes. This led to the hypothesis that, if bryophytes from contrasting habitats can have similar responses, desiccation tolerance (DT) is partially inductive and not only constitutive as previously proposed and, therefore, colony morphology might be the key trait responsible for controlling dehydration rate essential for DT induction. Morphology and life form may be determinant traits in the adaptation of bryophytes to habitats with different water availabilities and corresponding predicted levels in the DT inducibility spectrum. Bryophytes from habitats with different water availabilities were dried as individual shoots and as a colony. The bryophyte *Fontinalis antipyretica* is fully aquatic presenting a streamer life form, while the three terrestrial species present turf life form with different sizes and degrees of space between individuals in the colony. Two species were collected under trees with moist soil presenting short turf (*Tortella tortuosa*) and long turf (*Campylopus pyriformis*) life forms. Another species was completely exposed to sun light with no surrounding trees and a tall turf life form (*Pleurochaete squarrosa*). We used chlorophyll *a* fluorescence parameter F_v_/F_m_ (maximum potential quantum efficiency of Photosystem II) as a proxy to photosynthetic fitness throughout the contrasting dehydration rates (fast and slow). These bryophytes with different life forms were submitted to an X-ray computed microtomography (µ-XCT) to assess the three-dimensional inner structure and visualize locations for water storage. Shoots dried slow or fast according to the dehydration they were exposed to, as expected, but they presented similar dehydration rates across different species. However, the aquatic moss *F. antipyretica*, was unable to recover from fast drying, and after 24 h the recovery following slow drying was lower than the other species. The other three species presented full recovery after 24 h, either at the individual or colony level, and either from slow or fast drying. The only exception was the colonies of *Campylopus pyriformis* following fast drying that presented a slightly lower recovery, probably due to a looser colony structure.

## Introduction

In the past, bryophyte species have been classified in what concerns desiccation as either tolerant or sensitive (reviewed in Wood, 2007). Abel (1956) did one of the first attempts to classify vegetative desiccation tolerance (DT) in a wide range of bryophytes through vitality tests based on the exposure to a very wide range of relative humidity (RH) atmospheres (0 to 96% RH). These led to almost total desiccation of the tissues, but at very different desiccation rates. Some bryophytes, such as the aquatic species *Fontinalis antipyretica* only survived at 96% RH so it was classified as desiccation intolerant. Other authors reported similar conclusions based on measurements of photosynthesis in desiccated bryophyte tips exposed to fast dehydrating atmospheres (Lee and Stewart, 1971), electrolyte leakage (Brown and Buck, 1979) or habitat preference (Kimmerer and Allen, 1982; Seel et al., 1992; Franks and Bergstrom, 2000). Although the level of relative humidity at which these experiments were performed could be realistic, the rate of water loss (not monitored in these experiments) could be unrealistic, since these tests were made in isolated leaves or shoots, a condition that does not occur in nature. Most studies only followed recovery after rehydration over a few hours (Lee and Stewart, 1971; Brown and Buck, 1979). Some authors (Abel, 1956) had already pointed out briefly that some other aquatic bryophytes could develop DT under certain conditions; however, this was never the focus of attention of the present research in DT.

However, our recent works on *F. antipyretica* (Cruz de Carvalho et al, 2011; Cruz de Carvalho et al., 2012; Cruz de Carvalho et al., 2014; Cruz de Carvalho et al., 2015; Cruz de Carvalho et al., 2017) demonstrated that this bryophyte can cope with desiccation in certain conditions. The reason for the apparent contradiction in the DT classification of *F. antipyretica* in these recent works when compared with the previous ones (Abel, 1956; Lee and Stewart, 1971; Brown and Buck, 1979), could be related to the effect of different rates of water loss in bryophytes. However, this information was almost never present in previous works. The fact that the dehydration rate is important in DT is not new, but its relative importance was never fully evaluated across bryophytes. Some works quoted that only sensitive bryophytes needed slow dehydration prior to desiccation to become DT (Abel, 1956; Krochko et al., 1978). A close look to the data, and not to the conclusions reported on the previews works shows that even typical DT bryophytes such as the desert species *Syntrichia ruralis* and *Pterygoneurum lamellatum* need to be slowly dehydrated to avoid some type of damage (Schonbeck and Bewley, 1981; Stark et al., 2013).

Until recently, the few available works on proteins appear to indicate that no protein synthesis occurred during dehydration and DT bryophytes relied on constitutive protection coupled with a repair-based mechanism upon rehydration (Oliver, 1991; Wood and Oliver, 1999). However, works on proteomics in *Physcomitrella patens* and *F. antipyretica*. showed protein regulation during both dehydration and rehydration, being several proteins upregulated during dehydration, a clear indication of protein synthesis during this phase (Wang et al., 2009; Cui et al., 2012; Cruz de Carvalho et al., 2014). Moreover, it was demonstrated that desert bryophytes can lose their DT if kept hydrated and DT can be induced only during slow dehydration (Stark et al., 2013), refuting previous statements that DT bryophytes have only constitutive protection with the repair-based system operating following rehydration. Therefore, the rate of water loss is important for all bryophytes independently of the DT level (Cruz de Carvalho et al., 2011; Stark et al., 2013; Cruz de Carvalho et al., 2014; Cruz de Carvalho et al., 2017; Stark, 2017).

Dehydration rate can be classified as fast (less than an hour), slow (a few hours) or very slow (hours to days) (Oliver et al., 2004; Cruz de Carvalho et al., 2011; Stark et al., 2013). However, cellular dehydration rate is difficult to standardize across different bryophyte species (Alpert and Oliver, 2002), due to several parameters that influence dehydration rate such colony size and shoot morphology (Proctor, 2001; Elumeeva et al., 2011).

It is expected that bryophytes with different morphologies and life forms, dried in the same conditions and maintaining their initial tissue organization, will have different rates of dehydration at the cellular level. Therefore, desert bryophytes require morphologies and life forms that allow them to retain more water for longer periods. Most probably, bryophytes from habitats with higher moisture or even full aquatic ones have shoot morphology and colony life forms more adapted to other limiting factors than water such as nutrient interception. These colony life forms can be classified in cushions, short or tall turfs, mats, pendants, fans, dendroids and streamers (Gimingham and Birse, 1957; Mägdefrau, 1982; During, 1992; Glime, 2017) formed by shoots with different lengths and ramifications with axis supporting the leaves consisting of a single layer of cells. Therefore, shoot morphology and colony life form may be determinant traits in the adaptation of bryophytes to the different habitats that correspond to predicted levels of desiccation.

Since we cannot measure the rate of cellular dehydration, we can make use of techniques such as microscopy studies that provide interesting details to identify species and investigate several other aspects, including cell structure and ultrastructure. However, the analysis of complex matrixes such as bryophyte colonies interacting with environmental contexts in 3D qualitative and/or quantitative forms is hard to assess but is becoming of utmost importance. X-ray computed microtomography (µ-XCT) enables new qualitative and/or quantitative approaches in the study of shoot/colony morphology and life form exploring the matrix network textures (morphology, topology, geometry), ranging from macro- to microscopic spatial scales, in high-detailed 3D resolution (Maurício et al., 2013). The determination of colony water storage locations is important to understand how life form can control, to some extent, dehydration rate.

In the present work, we used the chlorophyll *a* fluorescence parameter F_v_/F_m_ (maximum potential quantum efficiency of Photosystem II) as a proxy to photosynthetic fitness throughout the contrasting dehydration rates in both shoots and colonies. Furthermore, we performed an exploratory analysis of dehydration rate and DT, using for the first time µ-XCT to assess the three-dimensional structure of different bryophyte morphologies, in colonies with different life forms, and assess with high detail water storage locations.

## Materials and Methods

### Plant Material

The aquatic bryophyte *Fontinalis antipyretica* Hedw. was collected in a shaded stream at Serra de S. Mamede National Park (39°16’07.4”N 7°18’56.4”W) and transported under cooling conditions (about 5°C) to the laboratory and cleaned in distilled water. The samples were kept in culture conditions, as previously described (Cruz de Carvalho et al., 2011), until assayed (one week). The terrestrial bryophytes *Pleurochaete squarrosa* (Brid.) Lindb., *Tortella tortuosa* (Hedw.) Limpr., and *Campylopus pyriformis* (Schultz) Brid. were collected in Mata da Machada National Forest (38°36’49.6”N 9°02’32.1”W) and transported in paper bags to the laboratory, allowed to dry and stored in dark and dry conditions until the assays (1 week).

For the assay of single shoots, each replicate (n = 3) consisted of a bundle of six shoot tips (*F. antipyretica* and *P. squarrosa*) or just one shoot (*T. tortuosa* and *C. pyriformis*) approximately with 1 cm length each.

For the assay of colonies, each replicate (n = 3) consisted of intact colonies with approximately 10 cm^3^ per replicate (*F. antipyretica* and *P. squarrosa*), and approximately 1.5 cm^3^ per replicate (*T. tortuosa* and *C. pyriformis*). In all assays, relative water content (RWC) was calculated according to Cruz de Carvalho et al. (2011).

### External Water Retention

The three terrestrial bryophytes were gently rehydrated with a hand sprinkler and allowed to recover for 3 days. Before the assays, samples were fully saturated by immersion in distilled water. Afterwards, all samples from the four species were removed from water, allowed for gravitational water drop and then weighted. Following blotting of external water (Cruz de Carvalho et al., 2015), samples were re-weighed, and external water retention was calculated as WR = (saturated weight/blotted weight).

### Dehydration Induction and Recovery

Since dehydration rate is influenced by several parameters tested in the current work (*e.g*., morphology, colony size) for the sake of simplicity, we use the terms slow and fast dehydration as referring to shoots dried in 95% relative humidity (RH) and 50% RH, respectively. This was attained by placing the samples in small containers over saturated salt solutions of K_2_SO_4_ (−6MPa) and Ca(NO_3_)_2_·4H_2_O (−100MPa), respectively. Replicates were kept in the same container for each separate dehydration rate assay. Throughout dehydration, samples were maintained under controlled temperature (approximately 20–23°C) and at low PAR (2–5 µmol m^−2^ s^−1^). Recovery from dehydration was made by rehydration in distilled water using a hand sprinkler.

### Chlorophyll a Fluorescence Analysis

Chlorophyll a fluorescence was measured prior to dehydration in order to determine the control values with a PAM 101 chlorophyll fluorometer (Heinz Walz GmbH, Effeltrich, Germany) connected to a PAM data acquisition system PDA 100 (Heinz Walz GmbH) and controlled by the software WinControl v2.08 (2003) (Heinz Walz GmbH). The maximum quantum efficiency of PSII (F_v_/F_m_), measured after dark adaptation, *i.e.*, when all PSII reaction centers are open (Baker and Oxborough 2005), was used as a measure of the bryophytes fitness since it decreases under dehydration stress (Cruz de Carvalho et al., 2011). To achieve this, samples were kept for 5 min in the dark, at the end of which a very weak measuring light pulse was turned on, to determine the basal fluorescence, F_o_, and afterwards a saturating light pulse (approximately 4,000 µmol m^−2^ s^−1^) (KL 2500 LCD, Schott AG, Mainz, Germany) was applied over the measuring light to determine the maximal fluorescence, F_m_. F_v_/F_m_, where F_v_ stands for variable fluorescence, was computed as (F_m_ − F_o_)/F_m_.

### X-ray Computed Microtomography Scanning and Processing

This study applies a method that has been developed for microfocal-based high-resolution systems (HRXCT) for sample monitoring and scanning in “static” dry/wet conditions. The method was designed and adapted to be usable on typical µ-XCT systems (Ketcham and Carlson, 2001; Cnudde and Jacobs, 2004; Ketcham and Iturrino, 2005; Bugani et al., 2007; Maurício et al., 2010). The µ-XCT device consists of the combination of an X-ray radiography microscopic scanner system and a computer controlling tomographic acquisition, reconstruction and analysis software packages (http://www.skyscan.be). Earlier research can be reviewed elsewhere (Ketcham and Carlson, 2001; Cnudde and Jacobs, 2004; Ketcham and Iturrino, 2005; Bugani et al., 2007; Maurício et al., 2010). The Skyscan 1172 contains an X-ray microfocus tube with high-voltage power supply, a specimen stage with precision manipulator, a 2-D X-ray CCD-camera connected to the frame grabber and a computer with color monitor. In order to obtain high-resolution images, small samples are preferred. X-ray source and detector are fixed, while the sample rotates around a stable vertical axis.

The plant material used for tomographic studies was selected as described in Section 2.1. Different approaches have been applied in plant preparation since immobility is required during tomographic acquisition step. All the samples were analyzed inside an Eppendorf tube to prevent further dehydration and/or movement. The aquatic bryophyte *F. antipyretica* Hedw. was gently blotted from external water with filter paper. A second acquisition was performed after drying the initial sample at room temperature. The terrestrial bryophytes *P. squarrosa* (Brid.) Lindb., *T. tortuosa* (Hedw.) Limpr., and *C. pyriformis* (Schultz) Brid. were first analyzed in dry state. The second/hydrated acquisition set was performed after water saturation inside Eppendorf tubes. The hydration process is apparently attained after few minutes of water supply, as can be seen in stereo microscope observations. Water excess was also removed from Eppendorf tubes by gentle blotting with filter paper.

The general procedure common to all µ-XCT scanning processes consists of the following: (i) acquisition; (ii) slice reconstruction; (iii) rendering; (iv) analysis of results (qualitative/quantitative) and (v) interpretation. Therefore, first, a series of views through the sample (*i.e.*, digital radiographs taken along different directions) are registered. Then, these views are recombined mathematically into a cross-sectional map (slice image) of the specimen’s X-ray absorptivity using microtomography instrumentation. The basic physical parameter quantified in each pixel of a CT-image is always the X-ray linear path intensity attenuation coefficient. Plant tissues exhibit different attenuation profiles, so it is very easy to distinguish the distinct tissues. To obtain 3-D information from the digital radiographic images produced by the µ-XCT, reconstruction software package had to be applied to those projection images. These projections were reconstructed using a modified Feldkamp cone-beam algorithm, and often a stack of about a thousand 2D cross section (slices) gray scale images representing the sample can be obtained. The images always contain a certain amount of noise that should be reduced. The X-ray microtomography operation procedure was optimized to produce the best images by reducing artefacts like beam hardening, ring, star and line artefacts as much as possible (Cnudde and Jacobs, 2004; Bugani et al., 2007; Maurício et al., 2010). Very dense sand particles, coexisting with terrestrial plants, can create a secondary radiation resulting in star artefacts. Ring artefacts appear as circles centered on the rotation axis and are caused by detector inaccuracies. To minimize ring artefacts, a random movement is applied to the object, together with its active area on the detector (Cnudde and Jacobs, 2004; Bugani et al., 2007; Maurício et al., 2010). For the aquatic bryophyte the acquisition conditions were: Source Voltage (40 kV); Source Current (250 µA); Image Pixel Size (4.16 µm); Rotation Step (0.3 deg/180 deg); Frame Averaging (3); Random Movement (3). For all the other species, the acquisition conditions were: Source Voltage (59 kV); Source Current (167 µA); Image Pixel Size (around 5 µm); Rotation Step (0.35 deg/180 deg); Frame Averaging (3); Random Movement (3). Four to six connected scans were performed with camera offset (×2 scanning size) with a total time of 2 h16 m acquisition for each scan.

All the tests included several bryophyte shoots in order to maximize collecting of information.

### Statistical Analysis

The statistical analyses were performed with GraphPad Prism 6.07 for Windows (2015) (GraphPad Software, San Diego California USA). For dehydration rate, it was applied the equation Y = Y0*exp(−K*X), where X is time, Y starts at Y0 (maximal value 100) and K is the rate constant applied. Three replicates of slow dehydrated and fast dehydrated samples were used in the measurements of dehydration rate assay. Whenever necessary, significant differences between groups were determined through ANOVA with Tukey post-test (significance level α = 0.05).

## Results

### External Water Retention

**Figure 1:**
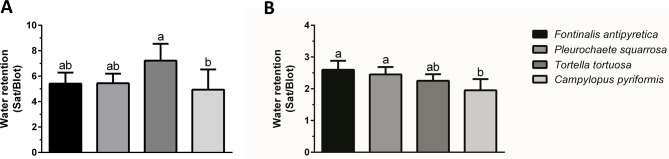
External water retention (saturated weight/blotted weight) at **(A)** individual shoots and **(B)** colony level in aquatic bryophyte (*Fontinalis antipyretica* Hedw.) and three terrestrial bryophytes (*Pleurochaete squarrosa* (Brid.) Lindb., *Tortella tortuosa* (Hedw.) Limpr., and *Campylopus pyriformis* (Schultz) Brid.). Different letters represent significant statistical differences (n = 3).

Determining the ability of bryophytes to retain water was compared by complete immersion of the colonies and individual shoots in water. Once removed from the water, and after excess water was removed by blotting, the observed external water retention was different depending on the structural level, colony or individual shoots (**Figure 1**). At the shoot level, water retained externally between 5 and 7-fold the water retained inside (**Figure 1A**), while at the colony level (**Figure 1B**), the retention was lower and with small variation between species (2 to 2.5-fold). Furthermore, at the shoot level, only statistically significant differences were observed between *T. tortuosa* and *C. pyriformis*, with the later showing lower water retention. Moreover, at the colony level differences were only observed between *C. pyriformis* and *F. antipyretica* (low DT) and *P. squarrosa* (high DT).

### Dehydration Rate *Versus* Colony Morphology and Size

**Figure 2:**
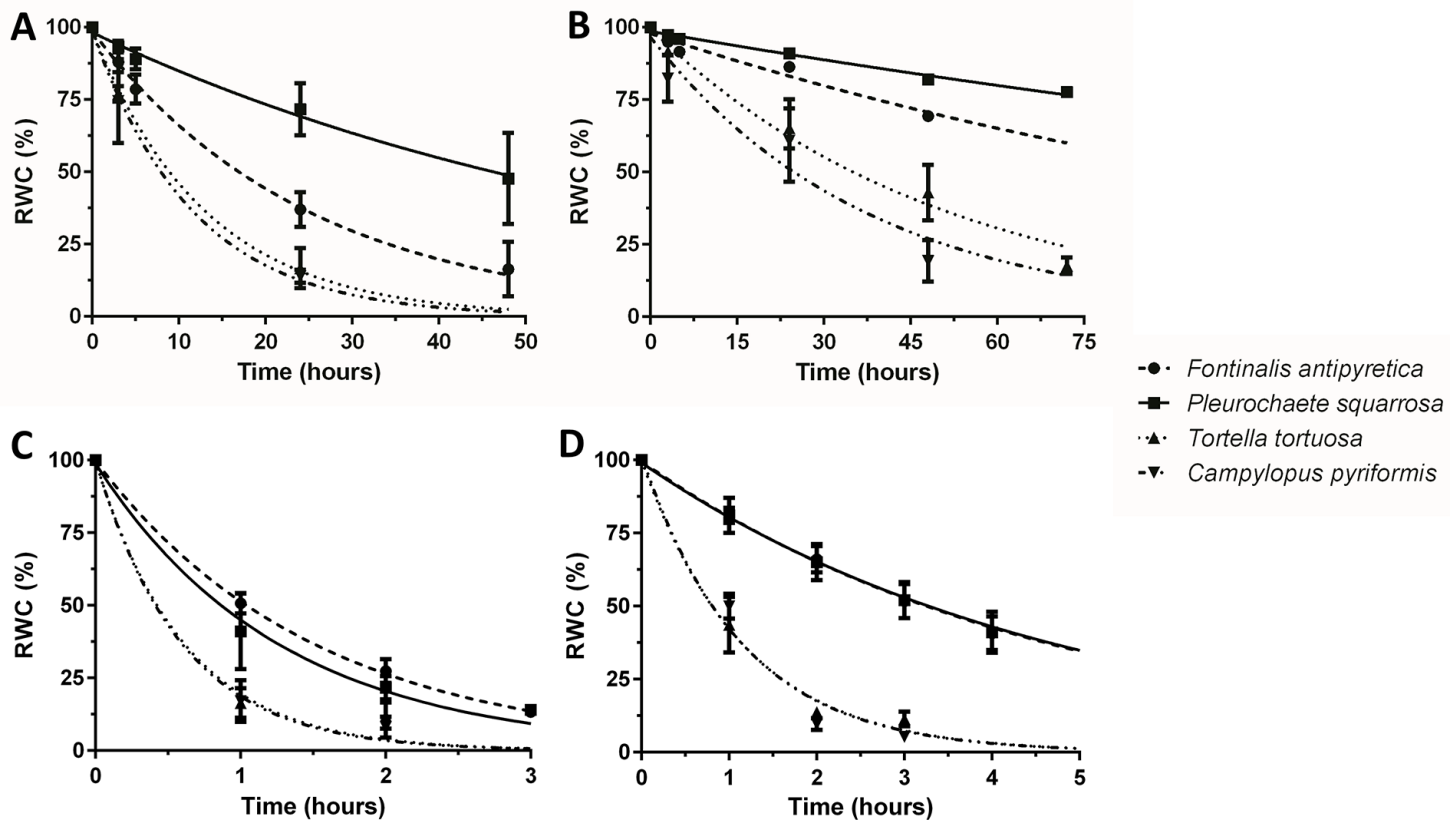
Dehydration rates under **(A**, **C)** fast and **(B**, **D)** slow drying in colonies **(A**, **B)** and shoots **(C**, **D)** of streamer life form bryophyte *Fontinalis antipyretica* Hedw. and turf life form bryophyte *Pleurochaete squarrosa* (Brid.) Lindb. (10 cm^3^ colonies; or six individual shoots), and in *Tortella tortuosa* (Hedw.) Limpr., and *Campylopus pyriformis* (Schultz) Brid.) with short and long turf life form (1.5 cm^3^ colonies; or one shoot) (n = 3).

Dehydration rate is influenced by several parameters, including size and life form of the colony. Therefore, we tested the mosses in two different dehydration rates. In the first assay (*F. antipyretica* vs *P. squarrosa*), we tested colonies of approximately the same volume (10 cm^3^) but very distinct morphology, with the aquatic moss presenting a streamer life form (long lawn-like), while the second develops as turf life form. *F. antipyretica* always presents a faster dehydration rate when compared with *P. squarrosa*, even more pronounced in the fast-drying conditions (**Figures 2A, B**). In the second assay (*T. tortuosa* and *C. pyriformis*), with smaller colonies (1.5 cm^3^) with short and long turf life form, respectively, the differences are less pronounced. Under fast drying, they dry at the same rate, while under slow dehydration there are differences, with *C. pyriformis* drying faster that *T. tortuosa* (**Figures 2A, B**). Nevertheless, the differences are striking when compared with *P. squarrosa* which has a much larger size although with similar morphology. Moreover, *F. antipyretica* colonies subjected to fast drying almost reach the rate of much smaller colonies of *T. tortuosa* and *C. pyriformis* (**Figure 2A**).

When the size of the colony decreases even further, down to six individual shoots (*F. antipyretica* and *P. squarrosa*) or even a single shoot (*T. tortuosa* and *C. pyriformis*) the colony effect is lost, with bryophytes being indistinguishable one from the other both in fast and slow dehydration (**Figures 2C, D**).

### Chlorophyll a Fluorescence Analysis

**Figure 3:**
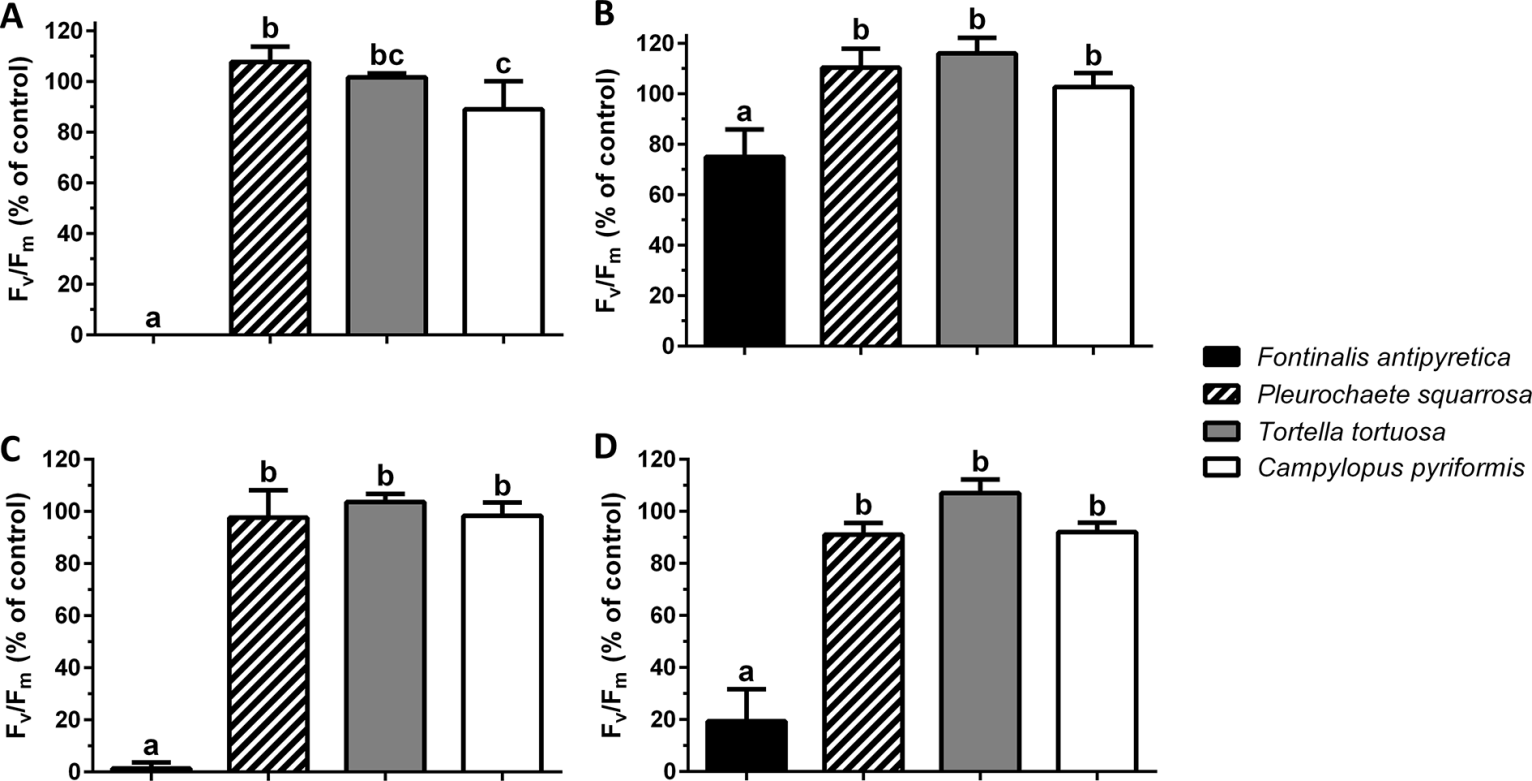
Maximum quantum efficiency of Photosystem II (F_v_/F_m_) (% of unstressed control) after 24 h rehydration following **(A**, **C)** fast and **(B**, **D)** slow drying in colonies **(A**, **B)** and shoots **(C**, **D)** of streamer life form bryophyte *Fontinalis antipyretica* Hedw. and turf life form bryophyte *Pleurochaete squarrosa* (Brid.) Lindb. (10 cm^3^ colonies; or six individual shoots), and in *Tortella tortuosa* (Hedw.) Limpr., and *Campylopus pyriformis* (Schultz) Brid.) with short and long turf life form (1.5 cm^3^ colonies; or one shoot) (n = 3).

At the colony level, colonies of *F. antipyretica* under slow drying show a recovery of 75% of unstressed control but not under fast drying which suggests a positive effect of the colony structure during dehydration (**Figure 3A**). The other three species presented full recovery after 24 h, either at the colony (**Figures 3A, B**) or individual (**Figures 3C, D**) level, at either slow or fast drying. Nevertheless, colonies of *C. pyriformis* following fast drying presented a slightly lower recovery (90% of unstressed control).

Regarding the maximum potential quantum efficiency of Photosystem II (F_v_/F_m_), at the shoot level and after 24 h the recovery, the aquatic moss *Fontinalis antipyretica*, was the only species unable to recover from fast drying (**Figures 3C, D**).

### X-ray Computed Microtomography Scanning Imaging

**Figure 4:**
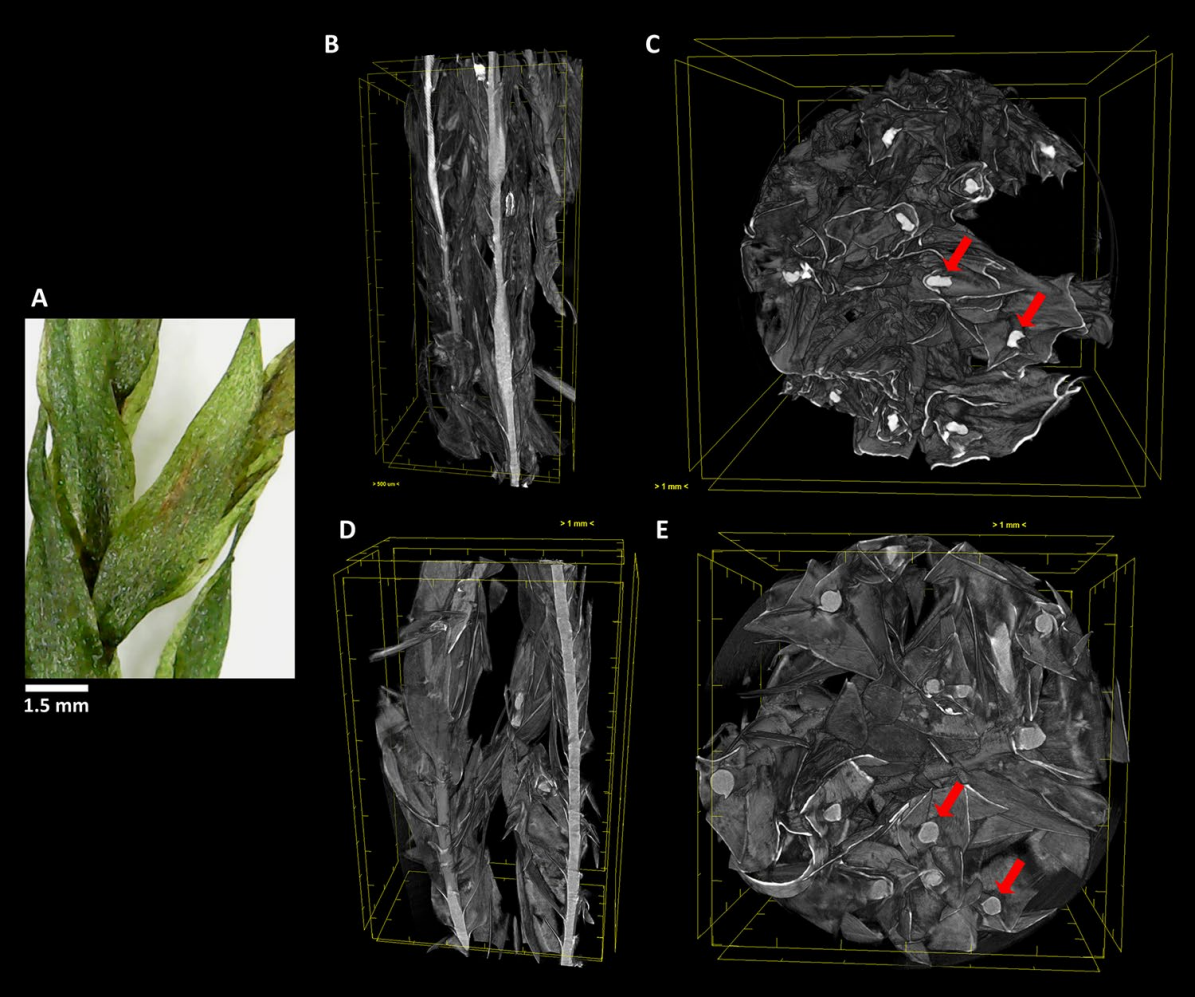
The aquatic bryophyte *Fontinalis antipyretica* Hedw. **(A)** visualized through µ-XCT scanning imaging of the colony structure **(B**, **D)** and transversal **(C**, **E)** cut, in dehydrated **(B**,**C)** and hydrated **(D**, **E)** states.

**Figure 5:**
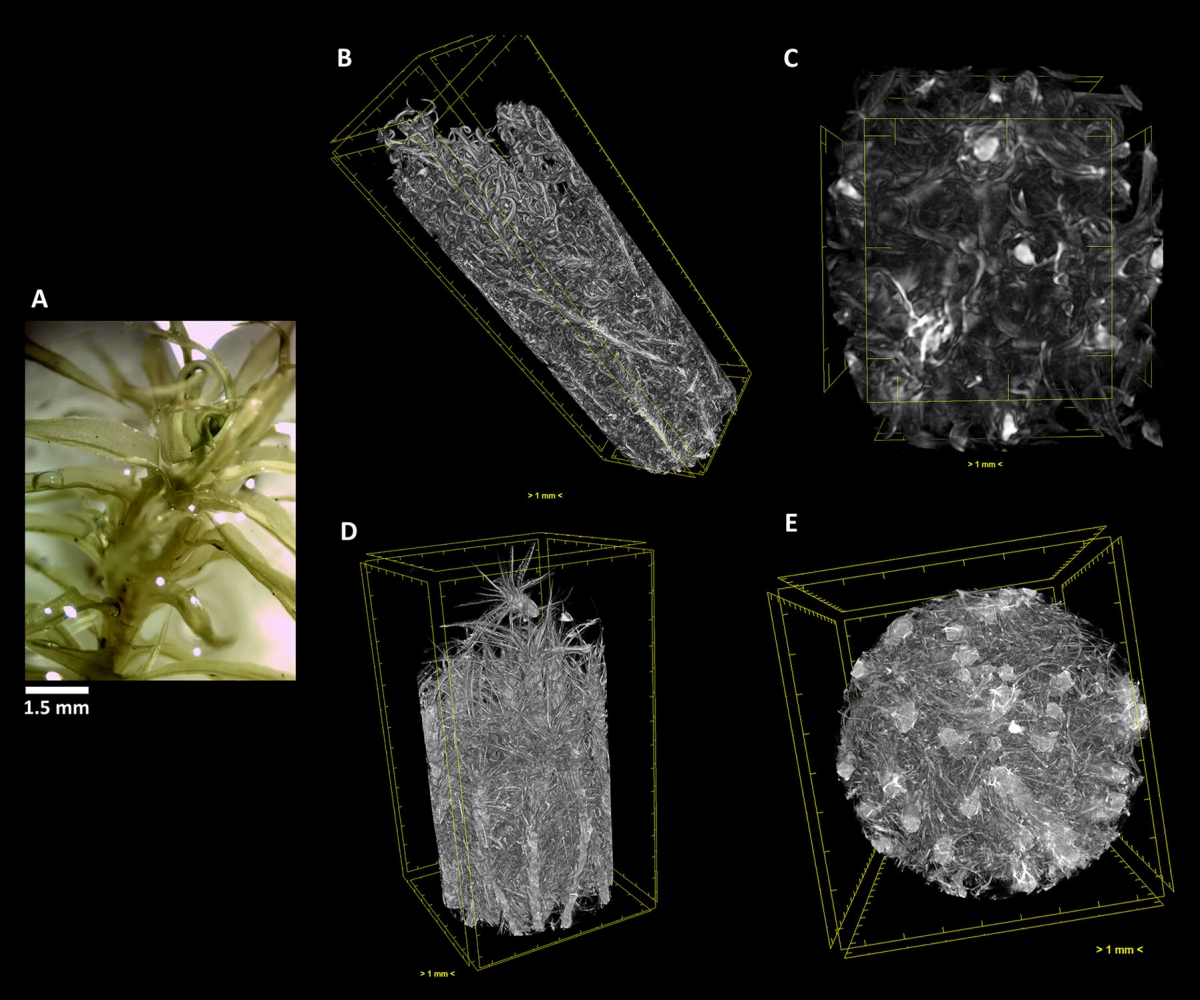
The terrestrial bryophyte *Pleurochaete squarrosa* (Brid.) Lindb. **(A)** Visualized through µ-XCT scanning imaging of the colony structure **(B**, **D)** and transversal **(C**, **E)** cut, in dehydrated **(B**, **C)** and hydrated **(D**, **E)** states.

**Figure 6:**
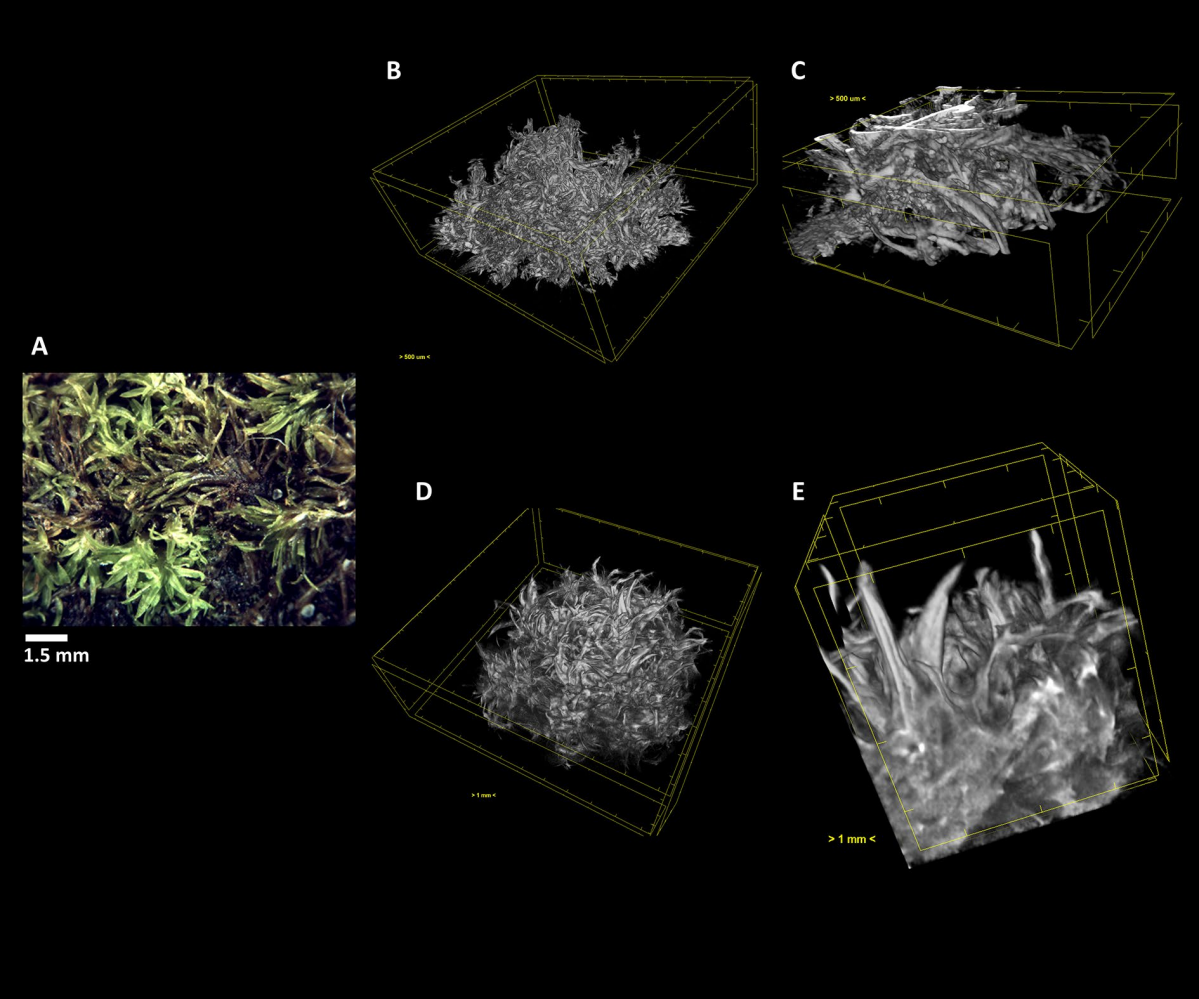
The terrestrial bryophyte *Tortella tortuosa* (Hedw.) Limpr. **(A)** Visualized through µ-XCT scanning imaging of the colony structure **(B**, **D)** and transversal **(C**, **E)** cut, in dehydrated **(B**, **C)** and hydrated **(D**, **E)** states.

**Figure 7:**
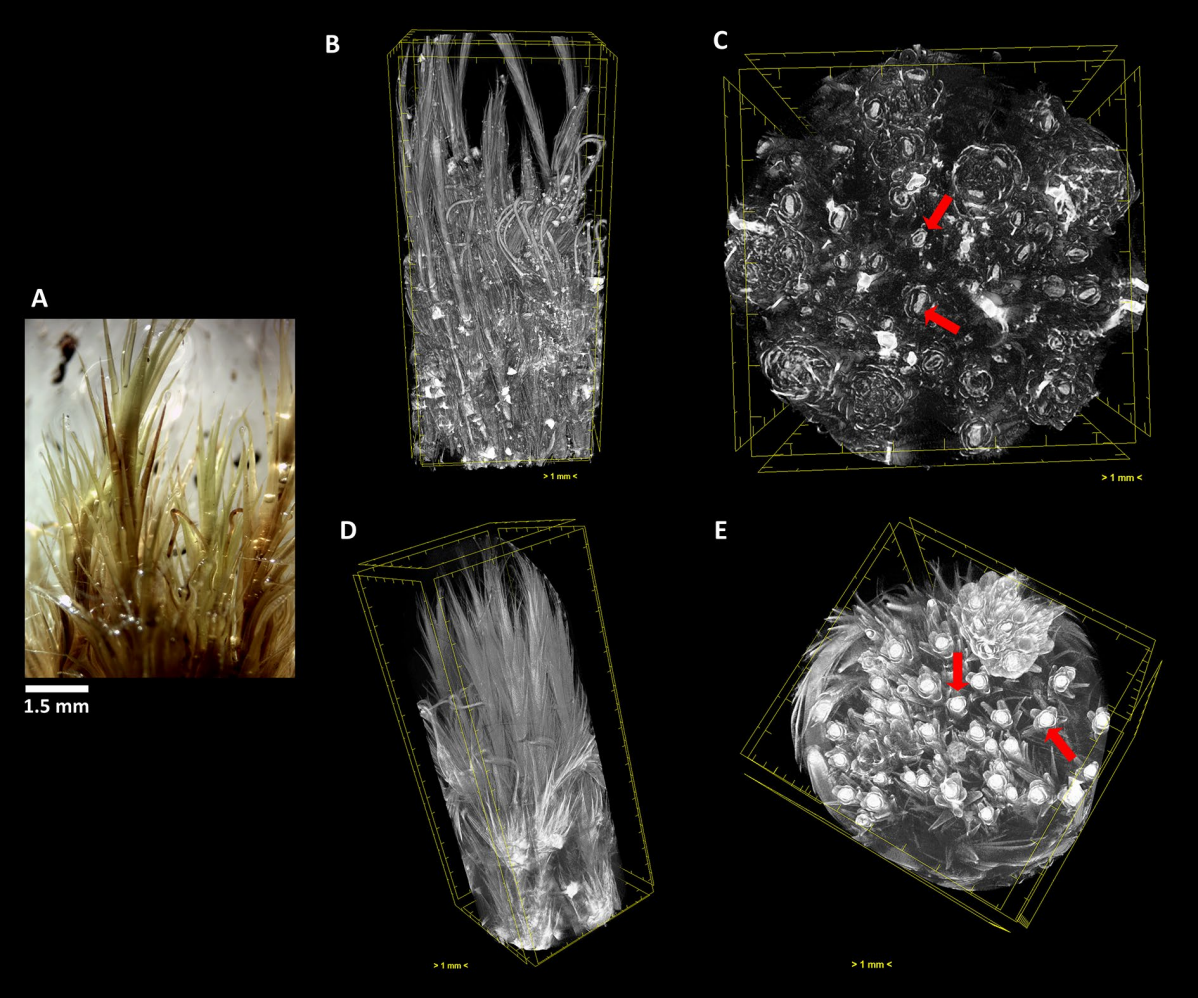
The terrestrial bryophyte *Campylopus pyriformis* (Schultz) Brid. **(A)** Visualized through µ-XCT scanning imaging of the colony structure **(B**, **D)** and transversal **(C**, **E)** cut, in dehydrated **(B**, **C)** and hydrated **(D**, **E)** states.

It is very advantageous that individual representative samples can be analyzed in distinct conditions by non-destructive methods during artificially induced environmental interactions. µ-XCT is one of these methodologies that can image the internal structure of visible light optically opaque samples, with spatial resolution of the same range used in optical microscopy. This technique allowed us to obtain extremely high details of the morphology and structure of the four analyzed bryophytes (**Figures 4**–**7**), both in the hydrated and dehydrated states. From these images, we can analyze any selected section and access water transport and storage.

In *F. antipyretica*, we can see the morphological change in the central axis of the cauloid which is collapsed in the dehydrated state, presenting an oval form (**Figure 4C**, red arrows) and resuming a spherical form upon rehydration (**Figure 4E**, red arrows), showing a clear increase in its volume. In the leaves the effected change is not so clear although the expansion upon rehydration led to an increase of the occupied volume (**Figures 4B, D**).

In *P. squarrosa*, the increase in volume of the leaves from the dehydrated (**Figures 5B, C**) to the fully hydrated state (**Figures 5B, E**) is visible, resuming its star-like morphology and with a complex network that is structured to maximize the water retention both in content and time.

In the short turf *T. tortuosa*, the central axis does not show significant changes in morphology, but the star-like leaves have a high volume increase from the dehydrated (**Figures 6B, C**) to the fully hydrated state trapping more easily the water (**Figures 6D, E**).

Similar to what occurred in *F. antipyretica*, in *C. pyriformis* the morphological change in the cauloid changed from being collapsed in the dehydrated state, presenting an oval form (**Figure 7C**, red arrows) to resuming the spherical form upon rehydration (**Figure 7E**, red arrows), showing a clear increase in its volume. The same increase in volume also is visible in the leaves presenting fine leaves in the dehydrated state (**Figure 7B**) to full expansion upon rehydration (**Figure 7D**), forming a fine network that allows to retain water for longer periods of time.

As we can see, this analysis can be performed in any section we choose, allowing to determine with high precision the amount of water that is absorbed going from the dehydrated to the hydrated states. Observing the transversal cuts, we observe a much more complex colony structural morphology in the turf life form (**Figures 5**–**7**), presenting a labyrinth-like appearance where water can be stored and more slowly released, when compared with the much looser morphology of *F. antipyretica* colony (**Figure 4**).

## Discussion

This work shows that bryophytes with contrasting habitats have different life forms which are an evolutionary response to the need to conserve water and in turn reflects their opposite position in the DT inducibility spectrum. However, among species with very similar life forms, shoot morphology and density contribute to their position in the spectrum. µ-XCT is a promising technique to reveal the 3D morphology in high detail, allowing to disentangle the structural complexity of the colonies’ life form and their position in the DT inducibility spectrum (Cruz de Carvalho et al., 2014). However, since during the process of dehydration/rehydration shoot movement occurs, the application of this technique still needs to be fine-tuned and this limitation should be addressed and minimized in future experiments.

During dehydration, photosynthesis shuts down (Hinshiri and Proctor, 1971; Dilks and Proctor, 1974; Tuba et al., 1996; Proctor et al., 2007a), high levels of soluble sugars occur in the cytoplasm (Bewley et al., 1978; Oldenhof et al., 2006), defense proteins increase (Alamillo et al., 1995; Wang et al., 2009; Cui et al., 2012), cytoskeleton is disassembled (Pressel et al., 2006; Wang et al., 2009) and sugar metabolism enzymes are up-regulated (Velasco et al., 1994; Wang et al., 2009; Cui et al., 2012). After rehydration, photosynthesis restarts (Tuba et al., 1996; Proctor and Smirnoff, 2000), cytoskeleton is re-assembled (Pressel et al., 2006) and high levels of soluble sugars (Bewley et al., 1978; Oldenhof et al., 2006), sugar metabolism enzymes and defense proteins (Cui et al., 2012) are maintained. In previous works, we showed that under field conditions *Fontinalis antipyretica* can survive the summer periods of Mediterranean drought (2–3 months) when the bryophyte is stranding out of water due to decreased river water flow (Cruz de Carvalho et al., 2011), inducing mechanisms of DT that are present in classical DT bryophytes (Cruz de Carvalho et al., 2012; Cruz de Carvalho et al., 2014; Cruz de Carvalho et al., 2015; Cruz de Carvalho et al., 2017).

Therefore, if bryophytes appear to be displaying similar physiological response patterns to desiccation, what is determining the different levels of DT in these organisms? We proposed that colony size and life form and shoot morphology may “control” the induction of such mechanism through differences in dehydration rates that allows time for induction and establishment of DT.

It is very difficult to compare the rate of dehydration across bryophyte species. One of the problems is their diverse morphological structures (Alpert and Oliver, 2002). As we have seen in this work, bryophytes with different life forms, dried in the same conditions and maintaining their initial colony organization, will dehydrate at completely different rates. Bryophytes from habitats with higher moisture or even aquatic will dry faster in a dry atmosphere because they have different life forms, more adapted to other limiting factors, such as withstanding the water current in a stream, as is the case of *F. antipyretica*, or water in the case of *Pleurochaete squarrosa*, *Tortella tortuosa* and *Campylopus pyriformis*.

On the other hand, bryophytes from drier habitats, such as the cases of *P. squarrosa*, *T. tortuosa* and *C. pyriformis*, are organized in more dense forms like cushions or mats. In denser bryophytes it is expected that higher water surface tension and water will be lost at lower rates than the less dense bryophytes. In fact, Proctor (1981), showed that surface tension was the most important factor. Thus, they naturally retain more water by capillarity and dehydrate more slowly than the others from damp locations when submitted to the same drying conditions maintaining the microclimate (gradients of humidity, temperature and wind speed) within the bryophyte, shaping its physiology and ecology under water deficit.

Through the µ-XCT imaging we showed the relation between the colony life form and morphology and the ability to retain water, therefore showing that the former is a determinant factor in the adaptation of bryophytes to each habitat, including to the prevailing levels of desiccation (Bates, 1998). More compact life forms (*e.g.* cushions) will reduce dehydration rate by modifying the microclimate, allowing time to develop DT mechanisms. This hypothesis is in accordance with the data suggesting that DT molecular mechanisms were developed early in evolution, upon land invasion in the Devonian (Kenrick and Crane, 1997), and therefore were present initially in all bryophytes. Under these circumstances, morphological adaptations might be the main evolution driver for adaptations to new habitats, according to water availability.

Fast dehydration has always a more deleterious effect on bryophyte recovery when compared with slow dehydration; this effect may result from the induction of protection/repair mechanisms. Slow dehydration allows even an aquatic bryophyte like *F. antipyretica* to be as tolerant as any xeric bryophyte (*e.g. P. squarrosa*, *T. tortuosa* and *C. pyriformis* in this work). Therefore, the mechanisms are kept active for some time allowing the preparation for drying. When plants are subjected to stress, sensing events connected to signaling cascades lead to restitution counter-reactions which, in turn, lead to the phase of resistance to stress. This sequence of events involves several molecular and structural processes, which require time to be implemented. This is similar to hardening, a well-known phenomenon in higher plants, common to different types of abiotic stress, which recent works have shown to also be present in bryophytes (Cruz de Carvalho et al., 2011; Cruz de Carvalho et al., 2014; Stark et al., 2013).

Although the question of whether the DT mechanisms are constitutive or inducible is still under debate (Proctor et al., 2007b), the evidence for a spectrum of DT and its inducibility appears as an increasingly more probable scenario (Cruz de Carvalho et al., 2014; Stark, 2017). In *S. ruralis*, an accumulation of mRNA during slow dehydration was observed and, apparently, no changes were found in proteins during this phase (Oliver, 1991; Wood and Oliver, 1999). However, the proteomic profile of this bryophyte during dehydration is still lacking to fully confirm this idea. As seen in Cruz de Carvalho et al. (2014) and other works (Wang et al., 2009; Cui et al., 2012) changes in proteins are very small but appear to be crucial for DT. Fast dehydration (sometimes in periods of only 30 min) may not allow the synthesis of new proteins, whereas slower dehydration rates will. The proportion and nature of these mechanisms may be variable, but DT is certainly not a characteristic determined only by constitutive protection mechanisms since some of these mechanisms can be induced by slow dehydration in other bryophytes. Therefore, dehydration rate is crucial to allow the establishment of DT mechanisms. The latter idea was confirmed in *F. antipyretica* (Cruz de Carvalho et al., 2011; Cruz de Carvalho et al., 2014), *P. patens* (Wang et al., 2009; Cui et al., 2012) and in the work by Stark et al. (2013) in the desert bryophyte *P. lamellatum* that lost DT after being kept hydrated for 5 days, after which fast drying was lethal. Moreover, DT was restored afterwards only by slow dehydration. Therefore, bryophytes need time to be prepared or induce some molecular synthesis in order to develop DT, which colony size and life form and shoot morphology have a very important role in controlling dehydration rate and inducing DT mechanisms. This knowledge will be of critical importance for the success in such scientific areas ranging from the ecological and landscape restoration using bryophyte-dominated biological soil crusts (Antoninka et al., 2015; Condon and David, 2016; Antoninka et al., 2018; Cruz de Carvalho et al, 2018) to the selection of bryophytes for green roofs (Brandão et al., 2017; Cruz de Carvalho et al., 2019; Paço et al., 2019).

## Conclusions

This work demonstrates that bryophyte morphology at shoot and colony levels is very important for DT and it might be the determinant factor in the adaptation of bryophytes to each habitat, leading them to respond in different ways to water availability. The technique of µ-XCT is a promising technique to help unravel this hypothesis, although, as this exploratory work showed, some limitations of the methodology, namely the shoot movements that occur during the dehydration/rehydration process, are still being perfected but eventually they will be addressed in future works.

*Fontinalis antipyretica* is under water most of the year and it presents higher vegetative growth rates (higher than 100-fold) compared with other terrestrial bryophytes like *S. caninervis* (Stark et al., 1998; Cruz de Carvalho, personal observation). On other hand, *S. caninervis* does not have much competition in its habitat while *F. antipyretica* must compete for resources with other aquatic macrophytes and microalgae. Thus, the investment in DT protection mechanisms *F. antipyretica* might be lower than the one in more xeric bryophytes, such as the Mediterranean species *P. squarrosa*, *T. tortuosa* and *C. pyriformis*. These species have a DT mechanism derived mostly from hardening due to exposure to daily fluctuations of water availability, but colony size and life form allows them to dry slowly over the morning period, remaining desiccated through most of the day. On the other hand, *F. antipyretica* may invest more on growth to better colonize the water streams. However, in Mediterranean streams, water level declines gradually during summer and, due to *F. antipyretica* life form, the long shoots overlapping each other, dehydration may be slow, allowing time for the induction of DT protective mechanisms. Once DT is established and the bryophyte completely dried, *F. antipyretica* can endure the dry season. Of course, the possibility of surviving a desiccation event during the summer, while other macrophytes dry out and die, may have an evolutionary advantage, since once the water becomes available, they can start to use the nutrients, while others are still germinating from spores or seeds. This also holds true for the terrestrial bryophytes, which benefit from a slow dehydration from the colony size and life form. Therefore, in the case of bryophytes, size (and form) does matter.

## Data Availability Statement

The datasets generated for this study are available on request to the corresponding author.

## Author Contributions

RC, JS, and CB conceived the ideas. RC, AM, and MP designed the methodology and acquired and analyzed the data. All authors contributed critically to the draft and the writing of the manuscript and gave final approval for publication.

## Funding

This study was supported by the PhD grant SFRH/BD/31424/2006 and the project MedMossRoofs (PTDC/ATPARP/5826/2014) both funded by the “Portuguese Foundation for Science and Technology (FCT)”, Portugal.

## Conflict of Interest

The authors declare that the research was conducted in the absence of any commercial or financial relationships that could be construed as a potential conflict of interest.
